# Intimate partner violence among older women in Reunion Island: an in-depth study of a cohort of 32 female victims from 2015 to 2025

**DOI:** 10.1192/j.eurpsy.2026.11569

**Published:** 2026-07-31

**Authors:** M. Pradinas, D. Doolub

**Affiliations:** 1 La Réunion, Centre Hospitalo-Universitaire La Réunion, Saint Pierre; 2 Vienne, Centre Hospitalier Henri Laborit, Poitiers, France

## Abstract

**Introduction:**

Intimate partner violence (IPV) represents a major public health concern in Réunion Island, yet no data exist regarding older women.

**Objectives:**

This study aims to explore the characteristics of IPV among older women in Réunion.

**Methods:**

We completed a retrospective case series study. Data was collected from the Medico-Legal Institute (IML) Unit at Félix Guyon University Hospital in Saint-Denis, Réunion. We extracted from the initial medical certificates issued between January 1, 2015, and June 3, 2025, among women aged 65 and older who are victims of IPV.

**Results:**

Thirty-two victims were included. All forms of IPV were identified: physical (87.5%), verbal (59.4%), psychological (62.5%), sexual (21.9%), and economic (12.5%), with four attempted femicide. Principal component analyses identified 2 distinct groups explaining 60.9% of the total variance (KMO = 0,5 ; *p* < 0,038) : factor 1 combining physical and verbal violence, and factor 2 grouping psychological, sexual, and economic violence, and associated with a shorter duration of IPV exposure (*β* = -0,60, *p* < 0,038, Nagelkerke *R^2^* = 0.288). We hypothesize that elderly women experiencing prolonged coercive control are less likely to seek formal assistance, or that an increase in coercive behaviors may occur with advancing age. The onset of violence occurred after the age of sixty-five for 31.3 % of victims. Longer histories of IPV were associated with the absence of separation (*β* = -0.98, *p* < 0,002) associated to the number of children (*β* = -0.98, *p* = 0,05); adjusted Δ*R^2^*=0.95. An alcohol use disorder was reported in 15.6% of the perpetrators. 71.9% of the women experienced physical consequences of IPV, with 15.6% suffering from severe acquired disabilities. Mental health repercussions were depressive symptoms for 53.1% of women, post-traumatic stress symptoms for 75% women, and 10.3% exhibited active suicidal ideation. Following the consultation, 43.8% of the women were referred for psychological or psychiatric care, and 13.3% were referred to a victim support association. Eighteen women were referred to the IML by the police, while only one patient was referred by the emergency department.

**Conclusions:**

Elderly women remain underrepresented in the IPV literature despite being exposed to severe violence with profound mental health consequences. Age at certification does not significantly influence characteristics or mental health impacts. Further research is needed to improve the care and support provided to these victims.

**Disclosure of Interest:**

None Declared

